# Is Infectious Endocarditis Evolving into a Time-Dependent Diagnosis in the Contemporary Epidemiological Era? Emphasis on the Role of Echocardiography as a First-Line Diagnostic Approach

**DOI:** 10.31083/j.rcm2410283

**Published:** 2023-10-08

**Authors:** Andrea Barbieri, Enrico Cecchi, Francesca Bursi, Francesca Mantovani

**Affiliations:** ^1^Division of Cardiology, Department of Diagnostics, Clinical and Public Health Medicine, Policlinico University Hospital of Modena, 41122 Modena, Italy; ^2^Department of Cardiology, Humanitas Cellini, 10100 Turin, Italy; ^3^Division of Cardiology, Dipartimento di Scienze della salute, San Paolo Hospital, ASST Santi Paolo and Carlo, University of Milan, 20142 Milano, Italy; ^4^Cardiology Unit, Azienda Unità Sanitaria Locale - IRCCS di Reggio Emilia, 42123 Reggio Emilia, Italy

**Keywords:** infective endocarditis, echocardiography, diagnosis

## Abstract

Despite significant advances in understanding and outcomes in various domains of cardiology, the prognosis of infective endocarditis (IE) remains dismal. One of the main reasons may rely on an even more intricate diagnosis since epidemiology has shifted towards an aggressive infection, typically in older patients with the involvement of prosthetic valves and cardiovascular implantable electronic devices with earlier clinical presentation. In this novel setting, it is critical to avoid a delay in diagnosis that may delay subsequent adequate treatment, further complications, and ultimately poor clinical outcomes. Accordingly, based on the available data, we will examine the proper use of first-line echocardiography representing the first-line imaging method in patients with clinical suspicion of IE. We will focus on the following three crucial questions: (1) What is the threshold to start the echocardiographic diagnostic workup in stable patients? (2) Has infective endocarditis become a time-dependent diagnosis, even in stable patients? (3) What is the appropriate use of echocardiography in unstable patients? Finally, we propose a new mindset to improve the echocardiographic diagnostic workflow.

## 1. Introduction

Despite advances in microbiologic, imaging diagnostic procedures and therapeutic management of infective endocarditis (IE), such as early surgery and targeted antibiotic therapy, the prognosis of IE remains dismal [1, 2]. One of the main reasons may rely on an even more intricate diagnosis since cases with “classical” clinical expression account for no more than 40% in the contemporary era [3]. The typical clinical signs of IE represent the hallmarks of subacute or chronic infections by indolent pathogens, such as viridans group streptococci traditionally seen in young patients with rheumatic heart disease. However, in developed countries, IE occurs increasingly as an acute disease with few of these hallmarks since epidemiology has shifted towards healthcare-associated IE due to virulent bacterial species infection such as *Staphylococcus aureus* causing an aggressive infection typically in older patients with involvement of prosthetic valves and cardiovascular implantable electronic devices with earlier clinical presentation [3, 4] and more frequent complications (transient ischemic attack or stroke) associated with higher in-hospital and one- year mortality [5]. Patient mortality from cardiac implantable electronic device (CIED) infections and IE is also significant [6], making early diagnosis and treatment critical in lowering death from this condition [7]. Given that the performance of the modified Duke criteria is far from ideal [8, 9], it is critical to avoid a delay in echocardiographic diagnosis that may delay subsequent adequate treatment, further complications, and ultimately poor clinical outcome [10]. On the other hand, underscoring the need for timely awareness of IE, many echocardiograms of little practical value are requested even in patients with low probabilities of IE, increasing the pressure that chronically affects the echo labs [11].

Accordingly, this narrative review summarizes the proper use of first-line echocardiography without jeopardizing the diagnosis of patients with possible and defined IE. We will focus on the following three crucial questions: (1) What is the threshold to start the echocardiographic diagnostic workup in stable patients? (2) Has IE become a time-dependent diagnosis, even in stable patients? (3) What is the appropriate use of echocardiography in unstable patients?

## 2. What is the Threshold to Start the Echocardiographic Diagnostic Workup in Stable Patients? 

Ideally, the primary diagnostic goal should be to minimize as much as possible the false negatives which require treatment. At the same time, transthoracic echocardiography (TTE) should not be utilized as a common screening tool for fever, considering the constraints on the echocardiography laboratory’s time and resources, but only in the presence of a reasonable clinical suspicion of IE [12]. Moreover, echocardiography should not be used as a stand-alone diagnostic tool but as part of a diagnostic strategy. Even if there is no quantitative data, it is a common clinical experience that systematic echocardiographic screening may increase the risk of false positive and false negative rates of IE diagnosis, overestimating the proportion of patients requiring complete IE therapy or provoking false reassurances with tangible downstream effects (Table 1). Notably, as echocardiographic technology improves, more subtle findings remain recognized and may exacerbate diagnostic uncertainty [13, 14].

**Table 1.** **Limitations of echocardiography in infective endocarditis: pitfalls**.

False positives risk	False negatives risk
•Filamentous structures: chordae rupture, sutures, strands	•Small vegetations (<2 mm)
•Advanced malignancy (marantic endocarditis)	•Non-vegetant IE (small leaflet thickening-early endocarditis)
•Tumors, valvular thrombus, lambl excrescences	•Not yet present (already embolized)
•Pre-existing lesions (MVP-myxomatous change, degenerative thickening/calcifications, AV cusp prolapse/laceration)	•Small abscesses (the earliest stage of disease, post-operative period, the presence of a prosthetic device esp. in the mitral position)
•Libman-Sachs vegetations	•Bad image quality, incomplete evaluation
•Old vegetations (usually echo-dense)	•Omission of TEE, omission of repeated exam
•Degeneration of a bioprosthesis, certain thrombi on valvular prostheses	•Omission of TEE in the prosthetic valve or intracardiac device

MVP, mitral valve prolapse; AV, aortic valve; IE, infective endocarditis; TEE, transesophageal echocardiography.

Defining a threshold for TTE diagnostic workup is challenging. Despite their extensive usage, the Duke criteria [15] do not indicate if echocardiography is required for all patients with “low clinical likelihood” because they lack a comprehensive set of defining criteria. A consensus has been reached, and transesophageal echocardiography (TEE) should be included in the diagnostic procedure in many cases of left-sided IE and intracardiac devices or prosthetic material [16] and when the clinical course is complicated by uncontrolled infection or heart failure [17]. However, TTE is always recommended to characterize the hemodynamic severity of valvular lesions, assess ventricular function and pulmonary pressures, and detect pericardial complications. When the ultrasound quality is sufficient, and there are no cardiac abnormalities that might increase the risk of IE or indicate an intracardiac infection (i.e., the absence of intracardiac catheters or other prosthetic material, abnormal valve anatomy or function, congenital cardiac abnormalities, pericardial effusion, and vegetation) TTE has been shown to provide a sufficient negative predictive value [18] and a subsequent confirmatory TEE is unnecessary [19]. Although there is no definition of what is technically adequate [20], a completely normal TTE result is more likely in patients with a low pretest probability (e.g., no heart murmur) but less common in patients with an intermediate or high pretest probability (e.g., prosthetic heart valve or acute valve regurgitation), who may still require TEE for its higher spatial resolution.

Echocardiography findings may be negative early in the disease course. Thus, repeated echocardiography is recommended in patients with negative initial echocardiography if high suspicion for IE persists [21]. In Europe, a relatively homogenous adherence to the current diagnostic echocardiographic recommendations in suspected IE has been observed [22] although, according to single-center research, several TEE examinations were deemed to be utilized improperly for IE [23].

A particular concern regards the still unsolved debate about the low enough risk of IE to justify the omission of TEE in *Staphylococcus aureus* bacteremia [24]. Currently, in Europe, most patients (92%) with *Staphylococcus aureus* bacteremia get an echocardiographic assessment at some point during their hospital stay: a TTE is followed by TEE in a quarter of the centers, and 18% used TEE as their initial diagnostic procedure [22].

The persistent mortality of IE leads to developing strategies for an early diagnosis of IE in patients with a bloodstream infection, which may help reduce complications and mortality of IE. A recent Danish nationwide registry that crossed the administrative data between blood cultures and the prevalence of IE over eight years indicates that echocardiographic screening for IE seems reasonable in patients with *Enterococcus faecalis*, *Staphylococcus aureus*, or *Streptococcus species* bacteriemia [25]. An alternative approach may be to develop multivariate predictive scores to estimate the risk of IE in patients with bloodstream infection. These scores are based on the setting of contraction of the infection (community vs. nosocomial, healthcare-associated non-nosocomial, or central-line-associated bacteremia), bacteremia duration, and the presence of intracardiac prosthetic material. In most of them, the persistence of bacteremia beyond 48–72 h and the high number of positive blood cultures are essential arguments in favor of IE diagnosis [26, 27, 28, 29]. The debate now focuses on which score performs best. The most precise estimate of the risk of IE in a very low-risk group of patients with *Staphylococcus aureus* bacteremia is currently conferred by a VIRSTA score of <3 points [29], Table 2 (Ref. [27]). The pooled negative likelihood ratio of the VIRSTA score is 0.08 (95% confidence interval 0.05–0.15). Based on the upper and lower bounds of the confidence interval, to have a post-test probability of <1.1% (i.e., below the proposed testing threshold), the pretest probability of IE should be <7% and <18%, respectively [30]. Therefore, no scoring system is still precise enough to place the patients below the testing threshold with 95% confidence, and none have been prospectively evaluated. The scores’ diagnostic accuracy may improve by incorporating additional parameters such as time to blood culture positivity [31].

**Table 2.** **VIRSTA Study**.

Factor	O.R.	Weight
Cerebral and/or peripheral embolization	10.4	5
Meningitis	9.6	5
Prior IE or permanent intracardiac device	7.3	4
Intravenous drug user	5.8	4
Persistent (>48 h) bacteriemia	3.9	3
Pre-existing native valve disease	3.6	3
Vertebral osteomyelitis	3.2	2
Community or non-nosocomial	2.6	2
Severe sepsis or shock	2.0	1
C-reactive protein >19 mg/dL	1.9	1

Multivariate Logistic Regression Model and Bootstrapping Procedure estimated the final predictive model of infective endocarditis in Staphylococcus aureus bacteriemia patients [27]. IE, infective endocarditis.

In general, it testifies against IE, the anamnestic contemporary absence of old age, bacteremia, cardiac murmur, drug addiction, predisposing heart disease, cardiac devices, diabetes, healthcare patients, embolic events, and immunosuppression [12].

## 3. Has Infective Endocarditis Become a Time-Dependent Diagnosis, Even in Stable Patients?

**Fig. 1.**
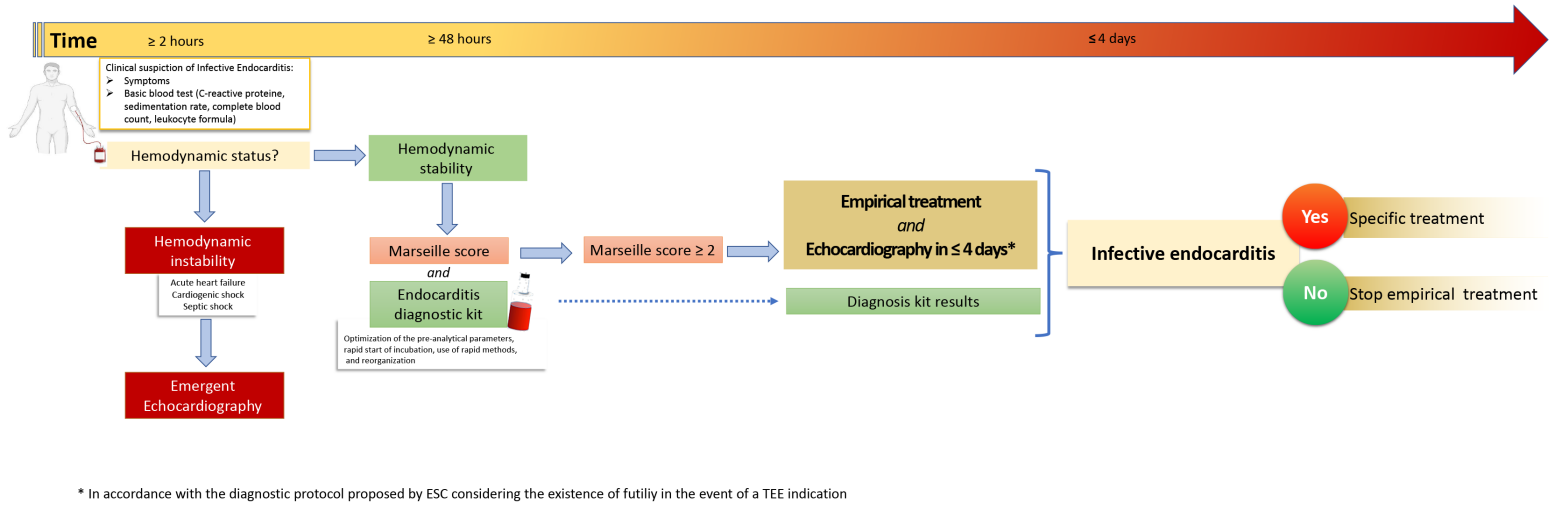
**Proposal of a new mindset and time-efficient diagnostic pathway to reach an improved diagnostic workflow focused on the early identification of related complications.** All rapid pathogen diagnostic methods need a 24/7 laboratory with skilled staff to maximize the positive effects. Blood culture remains the reference standard and first-line tool in the pathogen diagnostics of bloodstream infections and sepsis as the advantages of molecular technologies for rapid species identification have not yet been convincingly proved compared to the MALDI-TOF MS-based methods, which provide identification from positive blood culture in similar time for a more extensive range of microorganisms, with much lower cost for laboratories combined with rapid antimicrobial susceptibility testing. ESC, European Society of Cardiology; TEE, transesophageal echocardiography; MALDI-TOF MS, matrix-assisted laser desorption/ionization-time-of-flight mass spectrometry.

Analyzing the present recommendations [21, 32, 33, 34] the timing for echocardiography appears to be not critical, given that the guidelines’ maximum time limit varies from 5 to 10 days and does not yet explain how to recognize the clinical criteria warrant early echocardiography [35]. When IE is suspected, positive blood culture is unsuitable for rapid diagnosis, which can take several days to complete. Still, the prognosis of IE may be improved if people at high risk for IE can be identified early when such infection is suspected, hence shortening the time between suspicion, diagnostic echocardiography, and treatment and, thereby, the severity of valve destruction and complications related to IE [36]. With this goal, since 1994, any patient suspected of having IE who sought consultation or was admitted to one of the hospitals run by Assistance Publique of Marseille received testing using the diagnostic kit to be completed within two hours of hospital admission, mandating a battery of laboratory investigations, including three sets of blood cultures and systematic serological testing for *Coxiella burnetii*, *Bartonella spp*., *Aspergillus spp*., *Legionella pneumophila*, and rheumatoid factor. This standardization of etiological diagnosis processes, including thorough serologic testing, enabled 94% of all patients with definite IE to get an etiological diagnosis within five days [37]. Centered on these diagnostic kits, in 2008, Richet *et al*. [38] proposed, for patients with predisposing heart disease, the Marseille score, a very simple prediction tool to weigh and stratify the risk of IE based solely on criteria related to biological findings and clinical manifestations that are available or present upon admission (Table 3). When prospectively validated in an independent cohort of patients with clinical suspicion of IE [39], a score of 2 or more best predicted IE in patients with predisposing heart lesions. Sensitivity was better on left-side heart lesions (94%) than on right-side heart lesions (85%) (*p* = 0.04) and better for valvulopathy (94%) than intra-cardiac devices (84%) (*p* = 0.02). In addition, the positive predictive value of prosthetic valves was greater than that of native valves (*p* = 0.02). Therefore, this simple tool might determine when to expedite the first-line echocardiogram and begin empirical antibiotic medication after hospital arrival (Fig. 1). It is essential to recognize that the Marseille score has a not negligible percentage (8%) of false negatives, which could be even more significant in the contemporary epidemiological era were finger clubbing is rarely seen anymore. Therefore, patients should be constantly re-evaluated if a substantial clinical suspicion of IE persists. Furthermore, since 2008, when the diagnostic kit was first described in the Marseille score, numerous developments have been achieved (Fig. 1). Regarding analytical features, rapid diagnostic procedures utilizing cutting-edge technology have dramatically advanced [40]. Moreover, several molecular systems based on multiplexed polymerase chain reaction (PCR) or microarray have been suggested to directly detect and identify pathogens from positive blood cultures within 1–4 hours [41, 42], and the importance of the logistics and the improvement of quality management of blood culture [43] are increasingly recognized [44].

**Table 3.** **The Marseille score is determined during the first 24 hours of patient admission when a predisposing heart lesion is present**.

Marseille score on the day of admission
Male
Fever >38 °C
Peripheral arterial emboli
Stroke
Splenomegaly
Finger clubbing
Leukocytes >10.000/${\mathrm{mm}}^{3}$
Hemoglobin level <100 g/L
Erytrocyte sedimentation rate >50 mm or C reactive proteine >10 mg/L

According to the number of predictive factors identified by multivariate analysis, the score is derived by adding one point for each present parameter (range 0–9). Patients are divided into six score groups, with 0 representing no predictive factor and presenting six or more predictive variables.

Currently, no published studies link diagnostic echocardiography’s timing with outcomes in patients with suspected IE. Only one published study from St Bartholomew’s Hospital London evaluated the relation between time to definitive echocardiography (TTE or TEE if a definitive diagnosis was absent on TTE) and outcome [45]. This study showed that time to diagnostic echocardiography was an independent (albeit modest) predictor of valve destruction, and that late diagnostic echocardiography (≥four days) was a predictor of embolism during hospitalization. Overall, late diagnostic echocardiography was associated with a more significant requirement for valve surgery than patients receiving early diagnostic echocardiography. In this study, almost 40% of patients had to wait more than four days for a TTE. Remarkably, TTE and TEE were performed within four days of admission in only 62% and 15% of patients, respectively. Several factors may delay diagnostic echocardiography. For example, the diagnosis of IE may not be actively considered until late in the hospital course, or the patient may present with nonspecific sepsis, which may be mistaken for other pathologies.

## 4. What is the Appropriate Use of Echocardiography in Unstable Patients?

Hemodynamic instability is a well-recognized indicator of mortality risk in IE [1, 46, 47]. Infective endocarditis may present acutely with a rapidly progressive course complicated by acute heart failure (HF), cardiogenic shock (CS), severe sepsis, or septic shock (SS). SS and CS are not always distinct entities; conversely, there is some overlapping between the two. The emergence of SS is a strong determinant of mortality in IE [48]. Between 25–35% of people with IE present with acute HF (e.g., 27.2% in the European Society of Cardiology (ESC) EURObservational Research Programme (EORP) European Endocarditis (EURO-ENDO) registry) [1], 32.3% in the International Collaboration on Endocarditis–Prospective Cohort Study (ICE-PCS) cohort [47], and 34.7% in the ICE-Plus cohort [49]. The 2449 patients enrolled in the prospective ESC-EORP EURO-ENDO registry with left-sided (native or prosthetic) IE complicated by heart failure at the time of the IE diagnosis showed a significant excess risk of both 30-day mortality (20.5% vs. 9% in HF vs. non-HF) and 1-year mortality rates (36.1% vs. 19.3%) [50]. In a prospectively collected cohort from 35 Spanish centers (years 2008-2018), among 4856 IE patients, 34% had acute HF and 5% CS. Prosthetic valve IE accounted for 34% of CS cases. Roughly half of the patients experienced CS within 72 hours after being admitted for IE, with the other half developing it later during hospitalization (median of 4 days) [51]. More than half of CS cases were caused by mechanical reasons (valve regurgitation, peri annular complications, or pericardial tamponade).

It’s worth remembering that IE is the most common cause of acute regurgitation in a native or prosthetic aortic valve [52]. In patients with valvular IE, about one out of seven had multivalvular IE, mainly due to mitral-aortic involvement, associated with a poor in-hospital prognosis [53]. Acute mitral regurgitation and aortic regurgitation complicated by acute HF and CS are medical and surgical emergencies. In these patients, a rapid diagnosis is of the utmost importance, and appropriate therapy should not be postponed for diagnostic investigations that will not change the course of care considerably. Despite heterogeneity in etiology and valve position throughout the circulation, acute aortic and mitral regurgitation share some hemodynamic consequences: insufficient time for chamber adaption (remodeling) to additional blood volume, impaired forward stroke volume, the compensatory tachycardia, and the abrupt increase of pulmonary capillary wedge pressure [54, 55]. Since the cardiac examination findings of acute regurgitation differ from those of chronic regurgitation and are frequently less apparent, the diagnosis is often missed when a patient presents with severe dyspnea or an abnormal chest radiograph. Therefore, a high index of suspicion and the “disease-oriented” echocardiography is vital in rapid diagnosis. Another critical issue is differentiating CS from SS since the management is entirely different. In practice, this is not easy since some myocardial dysfunction frequently accompanies severe sepsis. In addition, CS and SS may sometimes co-exist, making the differential diagnosis problematic. Although natriuretic peptides would probably be helpful in the detection of early signs of acute heart failure in IE [56, 57] the disease-oriented echocardiographic diagnosis represents the mainstay for this differential diagnosis.

There are some principles to follow in patients with IE complicated by suspected acute valve regurgitation: (1) a thorough search for congruent anatomic lesions should be conducted, (2) frequently, point of care cardiac ultrasound (POCUS) modality is the first approach at the bedside, (3) the classical quantitative measures of severity are less valuable, (4) the color Doppler on TTE may underestimate regurgitation severity, mainly if the jet is eccentric, (5) the TEE is always needed. In patients with bacteriemia, for the detection of valvular vegetation, POCUS has sensitivity, specificity, and positive and negative predictive values of 77%, 94%, 82%, and 92%, respectively. For valvular regurgitation (more than mild), sensitivity is ≥76%, and specificity is ≥85% [58]. In acute regurgitation, measurements of effective regurgitant orifice area and regurgitant volume might be incorrect, especially if the patient is tachycardic. Hemodynamic studies have shown that the effective regurgitant orifice area and regurgitant volume vary depending on afterload and loading circumstances in acute regurgitation [59]. As a result, quantitative metrics seldom play a substantial role in treatment decisions in acute regurgitation. Instead, the vena contracta width measurement and density of the continuous wave Doppler signal are the most straightforward Doppler parameters to determine if significant regurgitation is present [54]. The TEE is often indispensable in identifying the tissue damage and, thus, the severity and mechanism of regurgitation if a TTE study is inconclusive, particularly with prosthetic valve dysfunction. However, if the diagnosis is obvious at the TTE and the decision is already pursued, the TEE can be done in the operating room. Due to its low temporal resolution, real-time three-dimensional (3D) TEE has fundamental limitations in tachycardic patients with hemodynamic instability [54].

**Fig. 2.**
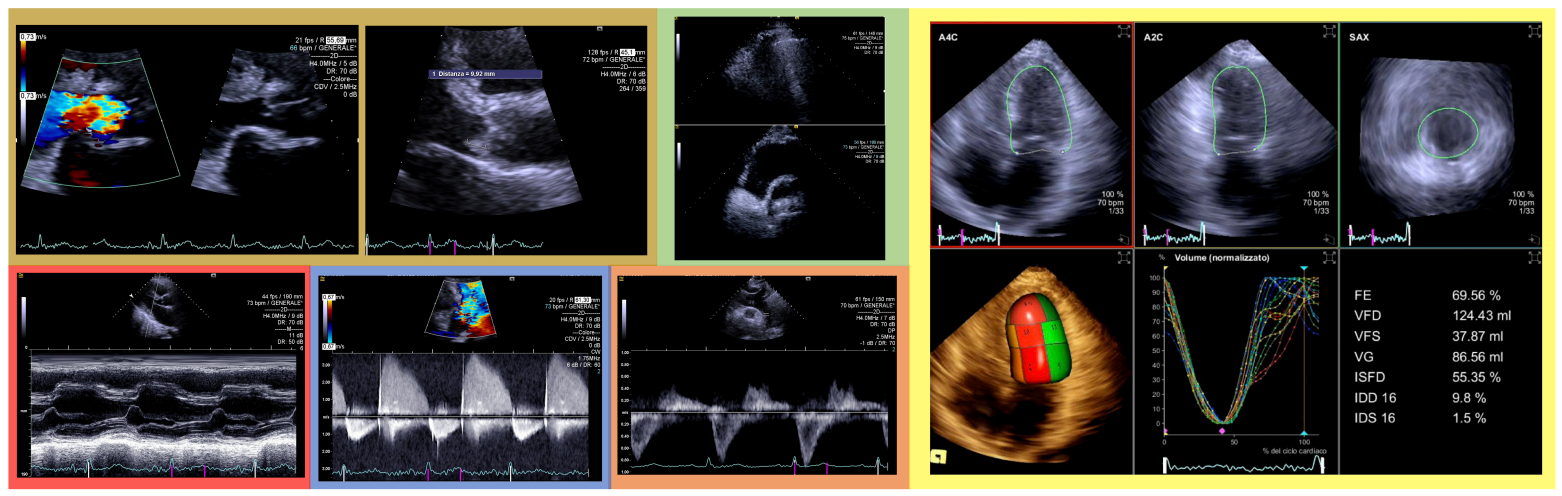
**Brown squares: on the left, Color Doppler shows aortic regurgitant jet width/left ventricular outflow tract that approaches 100% and vena contracta = 2 cm; on the right, vegetation adhering to the non-coronary cusp is evident with a maximum longitudinal diameter of 9 mm.** Green square: thoracic ultrasound demonstrates indirect signs of severe pulmonary congestion with a cardiogenic pattern (confluent b-lines in the upper part and pleural effusion in the lower position). Red square: M-mode echocardiography showing grade I early closure of the mitral valve with diminished A wave. Blue square: continuous-wave Doppler showing pressure half-time of 95 milliseconds. Orange square: pulsed-wave Doppler showing holo-diastolic flow reversal in the descending aorta. Yellow square: transthoracic real-time 3D demonstrates the absence of significant remodeling of the left ventricle (normal left ventricular volume: VFD) compatible with acute aortic valve regurgitation and an ejection fraction (FE) and stroke volume (VG) still within normal limits. 3D, three-dimensional; FE, ejection fraction; VFD, end-diastolic volume; VFS, end-sysyolic volume; VG, stroke volume; ISFD, end diastolic sphericity index; IDD 16, diastolic dyssynchrony index (16 segment model); IDS 16, systolic dyssynchrony index (16 segment model).

Premature mitral valve closure is a specific and sensitive noninvasive indicator of acute severe aortic regurgitation (Fig. 2). It has been correlated with the rise in left ventricular diastolic pressure [60]. Aortic valve surgery may be timed regarding whether the premature mitral valve closure is mild or severe. Some authors propose that aortic regurgitation (AR) patients exhibiting grade II premature mitral valve closure require urgent aortic valve replacement [61]. More generically, the ESC guidelines [21] stated that surgery is recommended in patients with severe acute AR who do not have clinical HF but have echocardiographic signs of elevated left ventricular end-diastolic pressure. This rule applies to both native valve IE and prosthetic valve IE.

## 5. Conclusive Remarks

Although the use of other imaging modalities, such as cardiac tomography and positron emission tomography, appears to be increasing [22], echocardiography remains a crucial first-line method to diagnose and consequently guide the management of IE in a timely-dependent fashion. Considering the contemporary epidemiological shift towards more aggressive pathogens and the complexity of the disease, its proper use should be increasingly time-efficient and focused on the early identification of associated complications or situations at risk of complications (e.g., large vegetations and severe regurgitation even without HF or CS) (Fig. 1). The time lag between echocardiographic diagnosis of IE and the onset of complications represents a potential window of opportunity for improving the still-unacceptable overall outcomes of IE. The path forward to better-identifying patients who need close monitoring and urgent surgery involves an earlier IE diagnosis, a transfer to referral centers with endocarditis teams, the identification of potential complication indicators, and performing aggressive treatment strategies in eligible patients where echocardiographic facilities are one the key components of a hub and spoke network [62]. Additionally, recognizing individuals at risk for CS and SS is crucial to distinguish between the two clinical scenarios since the first treatment approaches may differ significantly, particularly when administering intravenous fluids or when the surgical indication and timing are considered.

It is possible to identify at least five barriers that may delay diagnostic echocardiogram: local availability, inappropriate referrals, inadequate training of physicians, and omission of TEE/repeat exams. Regrettably, uncertainties remain regarding the appropriate testing threshold to start the echocardiographic diagnostic workup in stable patients. Based on currently yet limited evidence, it seems reasonable to expand rapid (<four days) access to echocardiographic testing in stable patients with predisposing heart lesions with Marseille score of 2 or more, mainly if the diagnostic kit detects *Enterococcus faecalis*, *Staphylococcus aureus*, or *Streptococcus species* bacteremia, given the high frequency of IE in such cases and the high morbidity and mortality associated with *Staphylococcus aureus* IE.

In patients without predisposing heart lesions, there is no question that clinicians should act on their appropriate clinical suspicion by ordering echocardiography as soon as possible, even if it does not satisfy the scoring requirements.

Furthermore, implementing matrix-assisted laser desorption/ionization-time-of-flight mass spectrometry (MALDI-TOF MS) and molecular approaches for species identification and rapid antimicrobial susceptibility testing can significantly reduce the time to result, with final species identification and antibiotic susceptibility testing report available within 24 hours of a positive bottle signal [63] (Fig. 1). Although the high variability in utility, dissemination, and cost of these new techniques makes defining the current standard of bloodstream infection pathogen diagnostics difficult, at least two meta-analyses have shown both cost-effectiveness and therapeutic improvements when rapid methods are in place [64, 65]. These paths should be formalized in a multidisciplinary program that allows obtaining some degree of reproducibility for diagnosis and treatment [66]. Likewise, the ESC guidelines [21] and the ACC/AHA statement claim that IE generally requires management by a team of physicians and allied health providers with various areas of expertise [32]. The relevance of the microorganism identification is further reinforced, considering the higher 30-day mortality in patients with culture-negative IE compared with patients with culture-positive IE. Interestingly, HF due to valvular dysfunction is more frequently observed in patients with culture-negative IE over the disease course [67, 68]. The diagnosis is based almost exclusively on imaging and primarily on echocardiography. Conversely, the endocarditis team approach has improved patient outcomes [69, 70]. Notwithstanding, two recent European surveys showed that the presence of an endocarditis team is still not dominant and comprises a specialist in echocardiography only in 2/3 of the cases [22, 71]. In contrast, there is no question that a fully accredited, competent echocardiography department is critical for the above reasons [72].

Our proposed timely first-line echocardiographic diagnostic strategy for IE should only be viewed as a hypothesis-generating proposal with the goal that it will be instructive for future research projects in this area. The major challenges are listed below:

- The prospective multicenter determination of its prognostic value in a larger cohort that includes secondary care unit;

- The demonstration that can help in the choice of the best therapeutic option: being more old patients with more prosthetic valves part of the cohort, a quick diagnosis would mainly be to assess for surgery, and since more are frail, surgery becomes less and less relevant;

- The assessment of its feasibility in different realities with different access to the imaging team within the endocarditis team;

- The integrated use of multi-modality imaging (computed tomography, magnetic resonance imaging, nuclear imaging) in different IE clinical scenarios: using multimodality imaging to identify cardiac and extracardiac IE-related lesions appears to be a promising strategy to aid in the care of patients with suspected IE. However, their use varies across countries, and their combinations are debated as much as current guidelines address the use of multimodality imaging in the field of IE with caution [73].

- The highest efficiency for each patient: in patients with CIED infection and IE, functional imaging with ^18^F-fluoro-2-deoxyglucose (FDG) positron emission tomography (PET) with computed tomography (CT) (FDG PET/CT) may have an incremental role in technically limited or inconclusive cases on echocardiography [74, 75].

There is still a long way to go in improving the time-efficient echocardiographic workup in patients with suspected IE and attaining a sufficient level of echocardiographic standardization across centers, both of which have potentially significant clinical implications in the modern epidemiological profile era. It is time for a broader discussion and possible consensus on the updates needed to improve present paradigms of echocardiographic assessment in IE.
